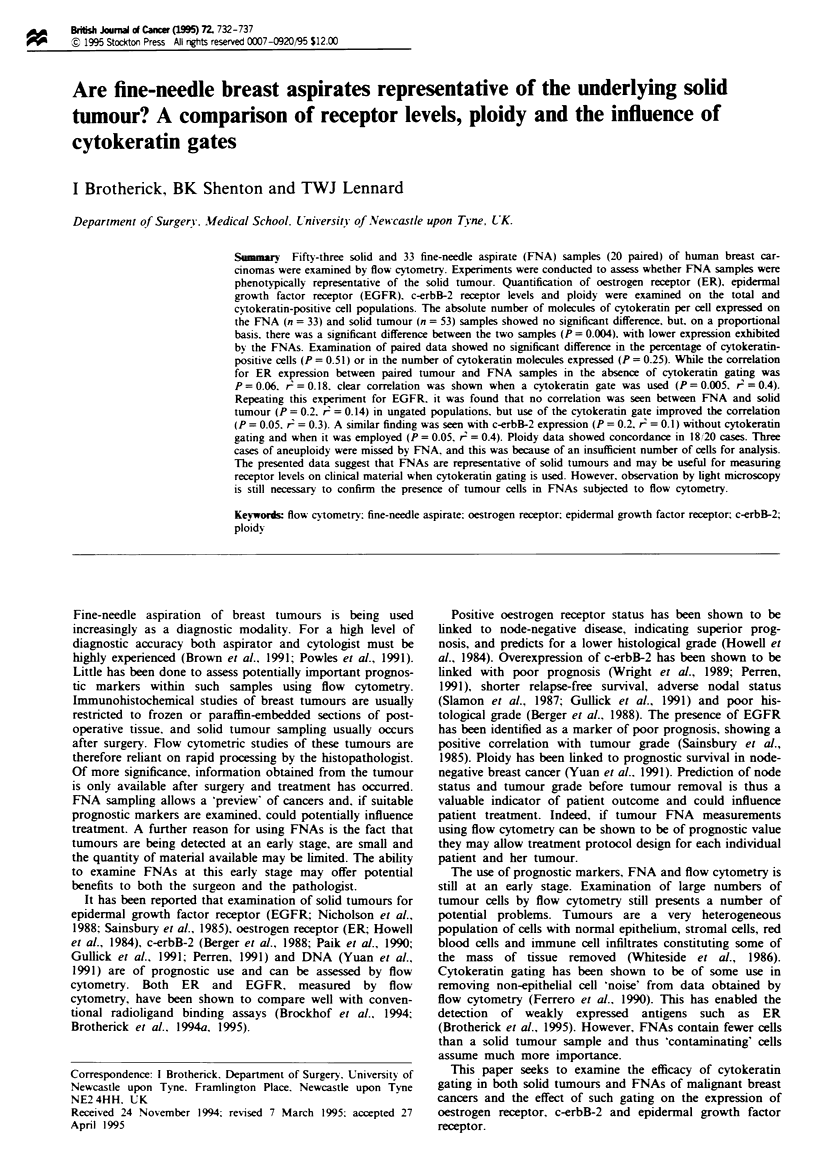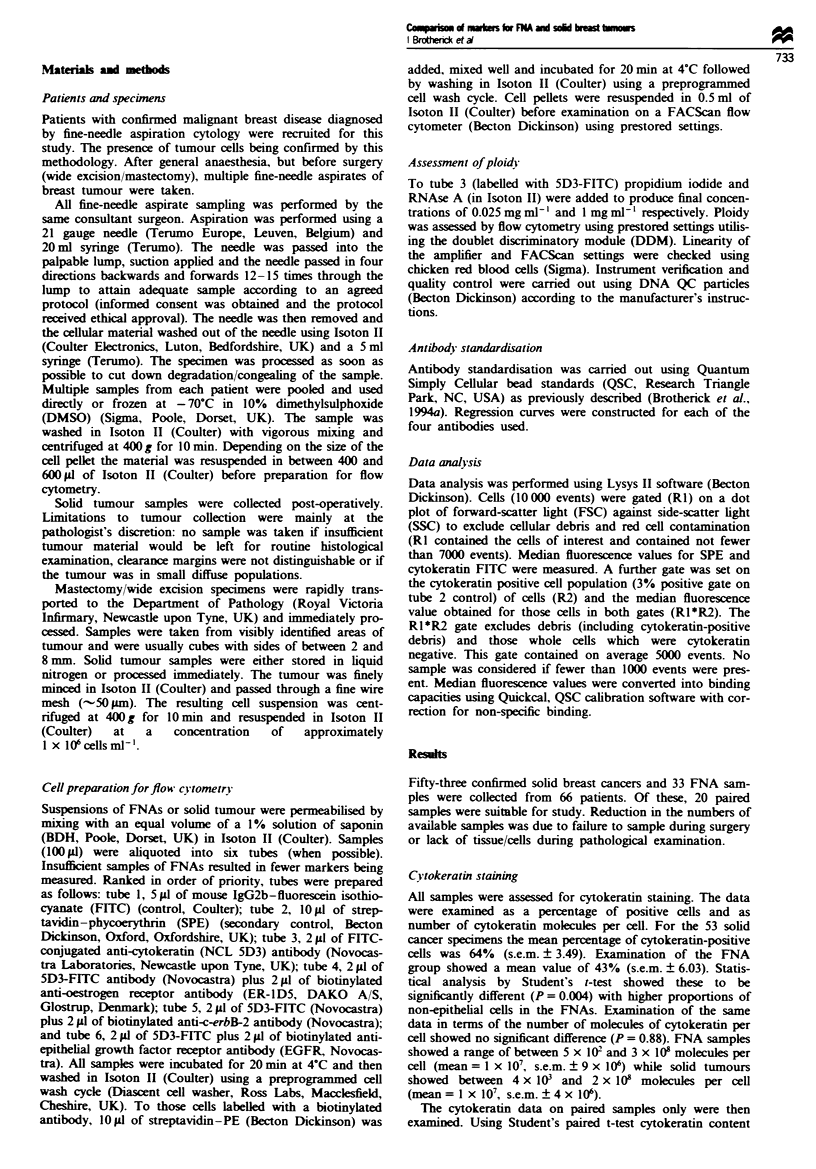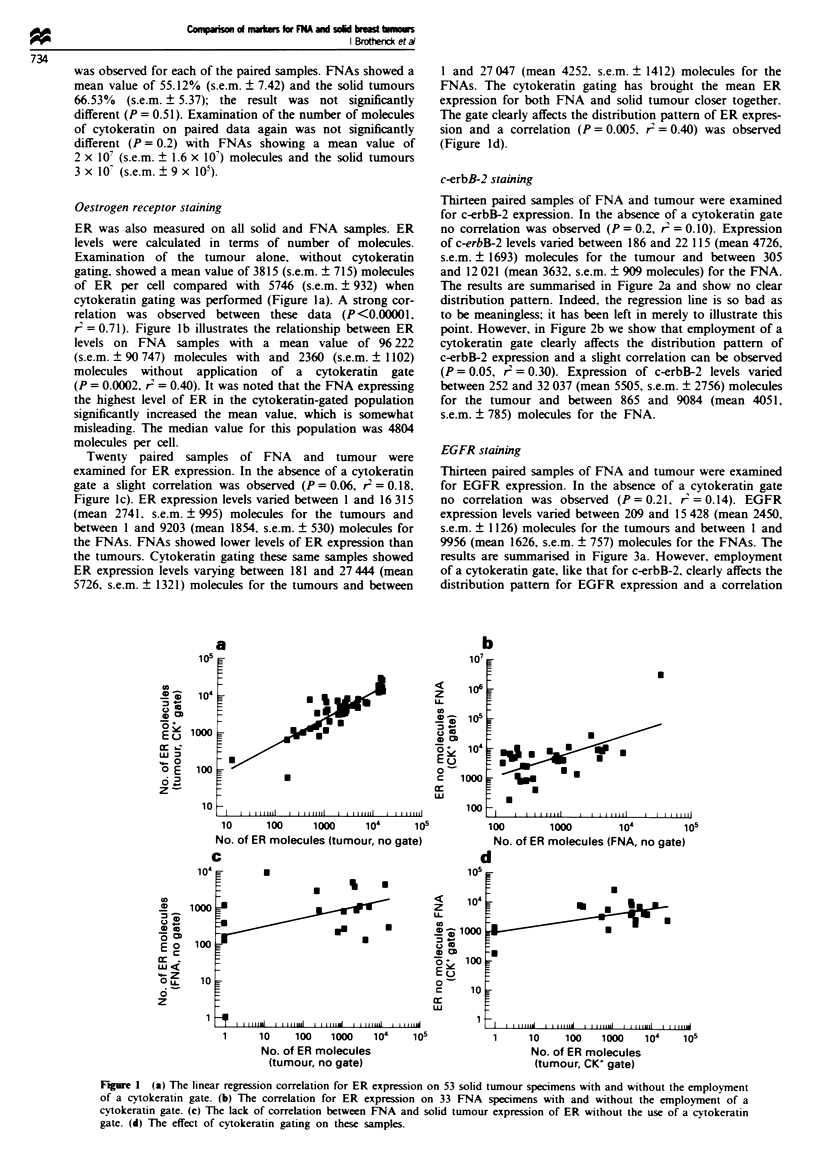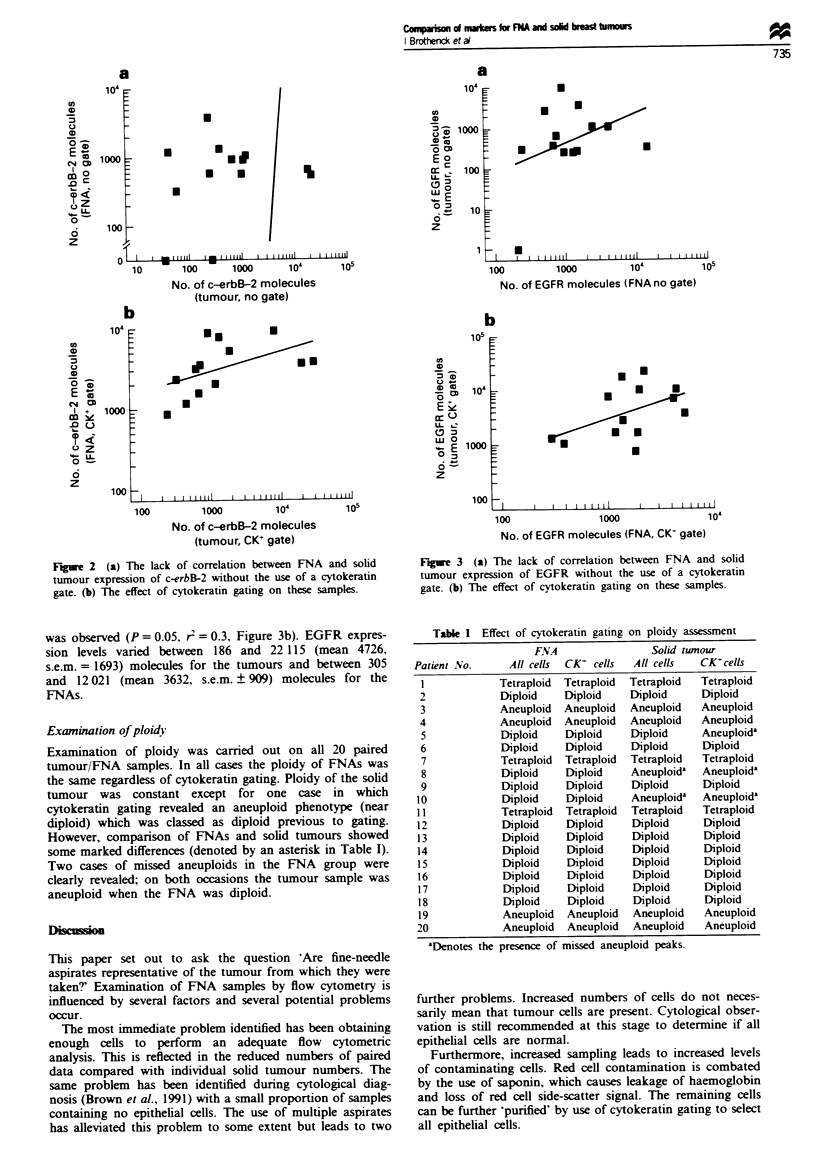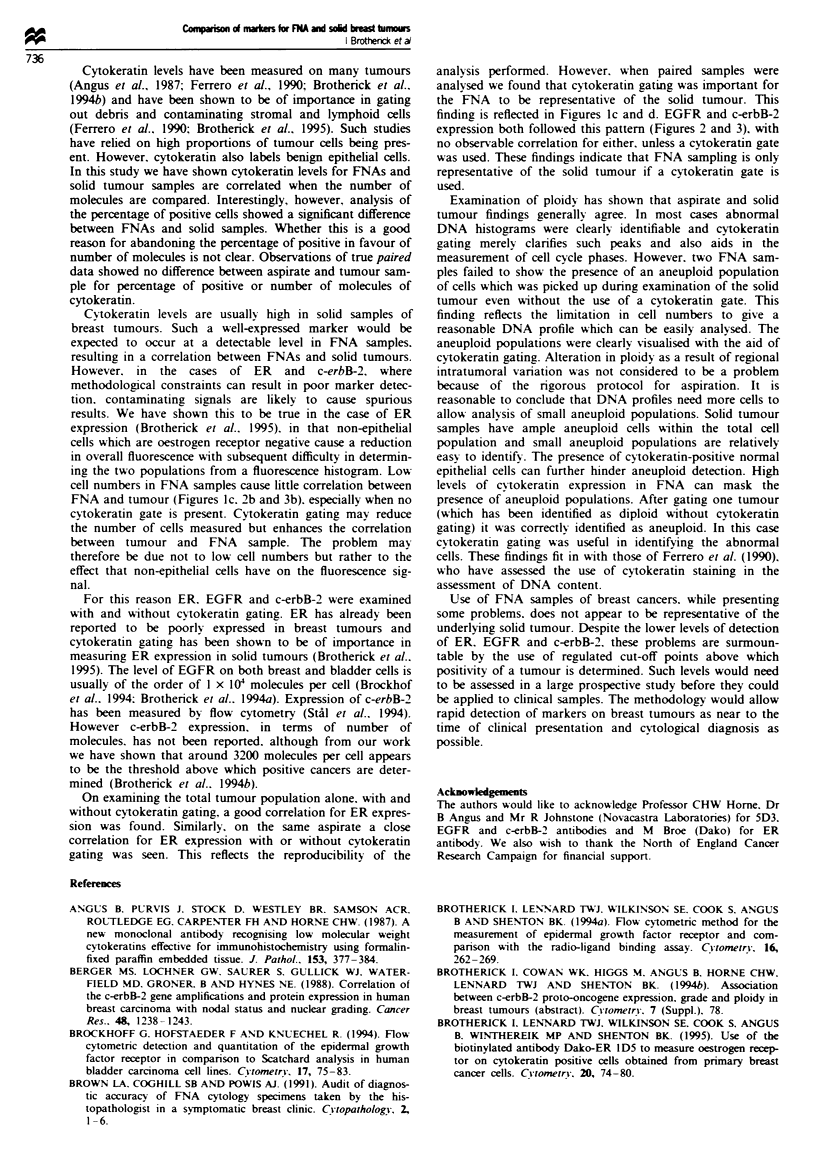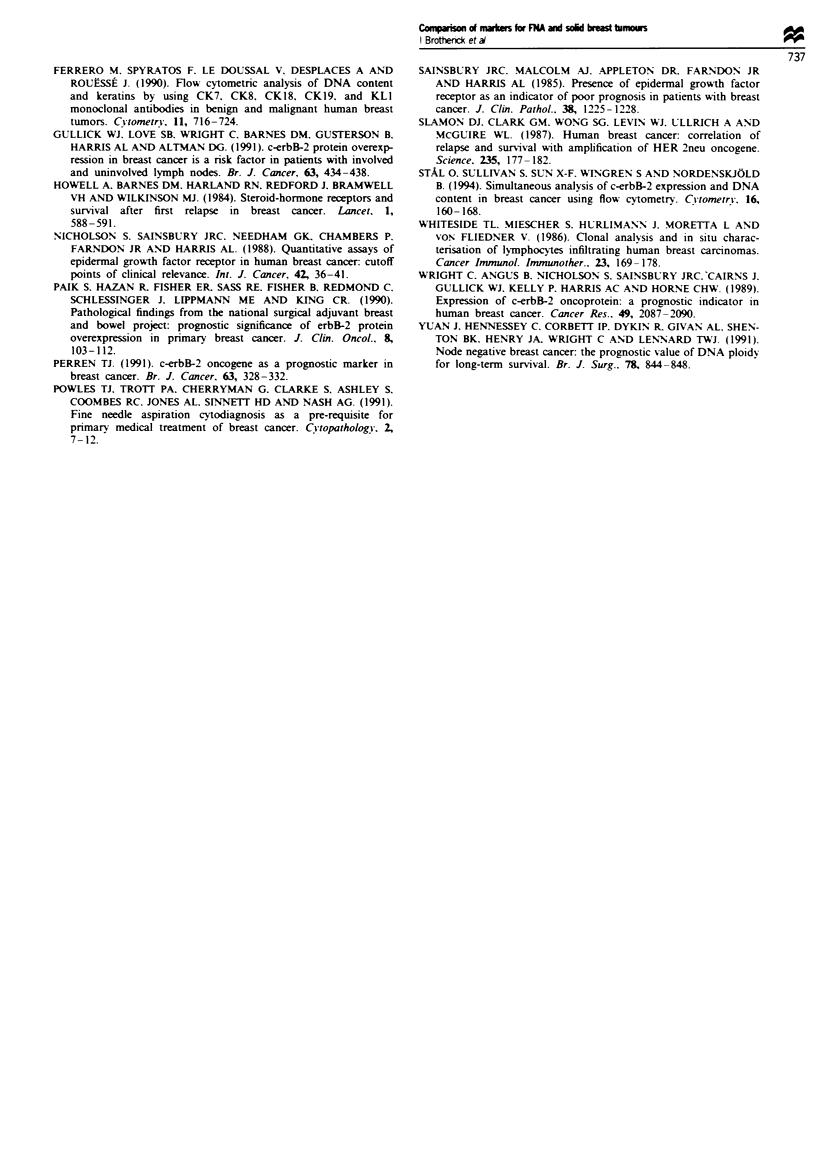# Are fine-needle breast aspirates representative of the underlying solid tumour? A comparison of receptor levels, ploidy and the influence of cytokeratin gates.

**DOI:** 10.1038/bjc.1995.402

**Published:** 1995-09

**Authors:** I. Brotherick, B. K. Shenton, T. W. Lennard

**Affiliations:** Department of Surgery, Medical School, University of Newcastle upon Tyne, UK.

## Abstract

Fifty-three solid and 33 fine-needle aspirate (FNA) samples (20 paired) of human breast carcinomas were examined by flow cytometry. Experiments were conducted to assess whether FNA samples were phenotypically representative of the solid tumour. Quantification of oestrogen receptor (ER), epidermal growth factor receptor (EGFR), c-erbB-2 receptor levels and ploidy were examined on the total and cytokeratin-positive cell populations. The absolute number of molecules of cytokeratin per cell expressed on the FNA (n = 33) and solid tumour (n = 53) samples showed no significant difference, but, on a proportional basis, there was a significant difference between the two samples (P = 0.004), with lower expression exhibited by the FNAs. Examination of paired data showed no significant difference in the percentage of cytokeratin-positive cells (P = 0.51) or in the number of cytokeratin molecules expressed (P = 0.25). While the correlation for ER expression between paired tumour and FNA samples in the absence of cytokeratin gating was P = 0.06, r2 = 0.18, clear correlation was shown when a cytokeratin gate was used (P = 0.005, r2 = 0.4). Repeating this experiment for EGFR, it was found that no correlation was seen between FNA and solid tumour (P = 0.2, r2 = 0.14) in ungated populations, but use of the cytokeratin gate improved the correlation (P = 0.05, r2 = 0.3). A similar finding was seen with c-erbB-2 expression (P = 0.2, r2 = 0.1) without cytokeratin gating and when it was employed (P = 0.05, r2 = 0.4). Ploidy data showed concordance in 18/20 cases. Three cases of aneuploidy were missed by FNA, and this was because of an insufficient number of cells for analysis. The presented data suggest that FNAs are representative of solid tumours and may be useful for measuring receptor levels on clinical material when cytokeratin gating is used. However, observation by light microscopy is still necessary to confirm the presence of tumour cells in FNAs subjected to flow cytometry.


British Journal of Cancer (1995) 72, 732-737

© 1995 Stockton Press All rights reserved 0007-0920/95 $12.00

### Are fine-needle breast aspirates representative of the underlying solid tumour? A comparison of receptor levels, ploidy and the influence of cytokeratin gates

I Brotherick, BK Shenton and TWJ Lennard

*Department of Surgery, Medical School, University of Newcastle upon Tyne, UK.*

**Summary** Fifty-three solid and 33 fine-needle aspirate (FNA) samples (20 paired) of human breast carcinomas were examined by flow cytometry. Experiments were conducted to assess whether FNA samples were phenotypically representative of the solid tumour. Quantification of oestrogen receptor (ER), epidermal growth factor receptor (EGFR), c-erbB-2 receptor levels and ploidy were examined on the total and cytokeratin-positive cell populations. The absolute number of molecules of cytokeratin per cell expressed on the FNA ($n = 33$) and solid tumour ($n = 53$) samples showed no significant difference, but, on a proportional basis, there was a significant difference between the two samples ($P = 0.004$), with lower expression exhibited by the FNAs. Examination of paired data showed no significant difference in the percentage of cytokeratin-positive cells ($P = 0.51$) or in the number of cytokeratin molecules expressed ($P = 0.25$). While the correlation for ER expression between paired tumour and FNA samples in the absence of cytokeratin gating was $P = 0.06$, $r^2 = 0.18$, clear correlation was shown when a cytokeratin gate was used ($P = 0.005$, $r^2 = 0.4$). Repeating this experiment for EGFR, it was found that no correlation was seen between FNA and solid tumour ($P = 0.2$, $r^2 = 0.14$) in ungated populations, but use of the cytokeratin gate improved the correlation ($P = 0.05$, $r^2 = 0.3$). A similar finding was seen with c-erbB-2 expression ($P = 0.2$, $r^2 = 0.1$) without cytokeratin gating and when it was employed ($P = 0.05$, $r^2 = 0.4$). Ploidy data showed concordance in 18/20 cases. Three cases of aneuploidy were missed by FNA, and this was because of an insufficient number of cells for analysis. The presented data suggest that FNAs are representative of solid tumours and may be useful for measuring receptor levels on clinical material when cytokeratin gating is used. However, observation by light microscopy is still necessary to confirm the presence of tumour cells in FNAs subjected to flow cytometry.

**Keywords:** flow cytometry; fine-needle aspirate; oestrogen receptor; epidermal growth factor receptor; c-erbB-2; ploidy

Fine-needle aspiration of breast tumours is being used increasingly as a diagnostic modality. For a high level of diagnostic accuracy both aspirator and cytologist must be highly experienced (Brown *et al.*, 1991; Powles *et al.*, 1991). Little has been done to assess potentially important prognostic markers within such samples using flow cytometry. Immunohistochemical studies of breast tumours are usually restricted to frozen or paraffin-embedded sections of post-operative tissue, and solid tumour sampling usually occurs after surgery. Flow cytometric studies of these tumours are therefore reliant on rapid processing by the histopathologist. Of more significance, information obtained from the tumour is only available after surgery and treatment has occurred. FNA sampling allows a 'preview' of cancers and, if suitable prognostic markers are examined, could potentially influence treatment. A further reason for using FNAs is the fact that tumours are being detected at an early stage, are small and the quantity of material available may be limited. The ability to examine FNAs at this early stage may offer potential benefits to both the surgeon and the pathologist.

It has been reported that examination of solid tumours for epidermal growth factor receptor (EGFR; Nicholson *et al.*, 1988; Sainsbury *et al.*, 1985), oestrogen receptor (ER; Howell *et al.*, 1984), c-erbB-2 (Berger *et al.*, 1988; Paik *et al.*, 1990; Gullick *et al.*, 1991; Perren, 1991) and DNA (Yuan *et al.*, 1991) are of prognostic use and can be assessed by flow cytometry. Both ER and EGFR, measured by flow cytometry, have been shown to compare well with conventional radioligand binding assays (Brockhof *et al.*, 1994; Brotherick *et al.*, 1994*a*, 1995).

Correspondence: I Brotherick, Department of Surgery, University of Newcastle upon Tyne, Framlington Place, Newcastle upon Tyne NE2 4HH, UK
Received 24 November 1994; revised 7 March 1995; accepted 27 April 1995

Positive oestrogen receptor status has been shown to be linked to node-negative disease, indicating superior prognosis, and predicts for a lower histological grade (Howell *et al.*, 1984). Overexpression of c-erbB-2 has been shown to be linked with poor prognosis (Wright *et al.*, 1989; Perren, 1991), shorter relapse-free survival, adverse nodal status (Slamon *et al.*, 1987; Gullick *et al.*, 1991) and poor histological grade (Berger *et al.*, 1988). The presence of EGFR has been identified as a marker of poor prognosis, showing a positive correlation with tumour grade (Sainsbury *et al.*, 1985). Ploidy has been linked to prognostic survival in node-negative breast cancer (Yuan *et al.*, 1991). Prediction of node status and tumour grade before tumour removal is thus a valuable indicator of patient outcome and could influence patient treatment. Indeed, if tumour FNA measurements using flow cytometry can be shown to be of prognostic value they may allow treatment protocol design for each individual patient and her tumour.

The use of prognostic markers, FNA and flow cytometry is still at an early stage. Examination of large numbers of tumour cells by flow cytometry still presents a number of potential problems. Tumours are a very heterogeneous population of cells with normal epithelium, stromal cells, red blood cells and immune cell infiltrates constituting some of the mass of tissue removed (Whiteside *et al.*, 1986). Cytokeratin gating has been shown to be of some use in removing non-epithelial cell 'noise' from data obtained by flow cytometry (Ferrero *et al.*, 1990). This has enabled the detection of weakly expressed antigens such as ER (Brotherick *et al.*, 1995). However, FNAs contain fewer cells than a solid tumour sample and thus 'contaminating' cells assume much more importance.

This paper seeks to examine the efficacy of cytokeratin gating in both solid tumours and FNAs of malignant breast cancers and the effect of such gating on the expression of oestrogen receptor, c-erbB-2 and epidermal growth factor receptor.

### Materials and methods

*Patients and specimens*

Patients with confirmed malignant breast disease diagnosed by fine-needle aspiration cytology were recruited for this study. The presence of tumour cells being confirmed by this methodology. After general anaesthesia, but before surgery (wide excision/mastectomy), multiple fine-needle aspirates of breast tumour were taken.

All fine-needle aspirate sampling was performed by the same consultant surgeon. Aspiration was performed using a 21 gauge needle (Terumo Europe, Leuven, Belgium) and 20 ml syringe (Terumo). The needle was passed into the palpable lump, suction applied and the needle passed in four directions backwards and forwards 12–15 times through the lump to attain adequate sample according to an agreed protocol (informed consent was obtained and the protocol received ethical approval). The needle was then removed and the cellular material washed out of the needle using Isoton II (Coulter Electronics, Luton, Bedfordshire, UK) and a 5 ml syringe (Terumo). The specimen was processed as soon as possible to cut down degradation/congealing of the sample. Multiple samples from each patient were pooled and used directly or frozen at −70°C in 10% dimethylsulphoxide (DMSO) (Sigma, Poole, Dorset, UK). The sample was washed in Isoton II (Coulter) with vigorous mixing and centrifuged at 400 *g* for 10 min. Depending on the size of the cell pellet the material was resuspended in between 400 and 600 μl of Isoton II (Coulter) before preparation for flow cytometry.

Solid tumour samples were collected post-operatively. Limitations to tumour collection were mainly at the pathologist's discretion: no sample was taken if insufficient tumour material would be left for routine histological examination, clearance margins were not distinguishable or if the tumour was in small diffuse populations.

Mastectomy/wide excision specimens were rapidly transported to the Department of Pathology (Royal Victoria Infirmary, Newcastle upon Tyne, UK) and immediately processed. Samples were taken from visibly identified areas of tumour and were usually cubes with sides of between 2 and 8 mm. Solid tumour samples were either stored in liquid nitrogen or processed immediately. The tumour was finely minced in Isoton II (Coulter) and passed through a fine wire mesh (~50 μm). The resulting cell suspension was centrifuged at 400 *g* for 10 min and resuspended in Isoton II (Coulter) at a concentration of approximately $1 \times 10^6$ cells $ml^{-1}$.

*Cell preparation for flow cytometry*

Suspensions of FNAs or solid tumour were permeabilised by mixing with an equal volume of a 1% solution of saponin (BDH, Poole, Dorset, UK) in Isoton II (Coulter). Samples (100 μl) were aliquoted into six tubes (when possible). Insufficient samples of FNAs resulted in fewer markers being measured. Ranked in order of priority, tubes were prepared as follows: tube 1, 5 μl of mouse IgG2b–fluorescein isothiocyanate (FITC) (control, Coulter); tube 2, 10 μl of streptavidin–phycoerythrin (SPE) (secondary control, Becton Dickinson, Oxford, Oxfordshire, UK); tube 3, 2 μl of FITC-conjugated anti-cytokeratin (NCL 5D3) antibody (Novocastra Laboratories, Newcastle upon Tyne, UK); tube 4, 2 μl of 5D3-FITC antibody (Novocastra) plus 2 μl of biotinylated anti-oestrogen receptor antibody (ER-1D5, DAKO A/S, Glostrup, Denmark); tube 5, 2 μl of 5D3-FITC (Novocastra) plus 2 μl of biotinylated anti-c-*erb*B-2 antibody (Novocastra); and tube 6, 2 μl of 5D3-FITC plus 2 μl of biotinylated anti-epithelial growth factor receptor antibody (EGFR, Novocastra). All samples were incubated for 20 min at 4°C and then washed in Isoton II (Coulter) using a preprogrammed cell wash cycle (Diascent cell washer, Ross Labs, Macclesfield, Cheshire, UK). To those cells labelled with a biotinylated antibody, 10 μl of streptavidin–PE (Becton Dickinson) was added, mixed well and incubated for 20 min at 4°C followed by washing in Isoton II (Coulter) using a preprogrammed cell wash cycle. Cell pellets were resuspended in 0.5 ml of Isoton II (Coulter) before examination on a FACScan flow cytometer (Becton Dickinson) using prestored settings.

*Assessment of ploidy*

To tube 3 (labelled with 5D3-FITC) propidium iodide and RNAse A (in Isoton II) were added to produce final concentrations of 0.025 mg $ml^{-1}$ and 1 mg $ml^{-1}$ respectively. Ploidy was assessed by flow cytometry using prestored settings utilising the doublet discriminatory module (DDM). Linearity of the amplifier and FACScan settings were checked using chicken red blood cells (Sigma). Instrument verification and quality control were carried out using DNA QC particles (Becton Dickinson) according to the manufacturer's instructions.

*Antibody standardisation*

Antibody standardisation was carried out using Quantum Simply Cellular bead standards (QSC, Research Triangle Park, NC, USA) as previously described (Brotherick *et al.*, 1994*a*). Regression curves were constructed for each of the four antibodies used.

*Data analysis*

Data analysis was performed using Lysys II software (Becton Dickinson). Cells (10 000 events) were gated (R1) on a dot plot of forward-scatter light (FSC) against side-scatter light (SSC) to exclude cellular debris and red cell contamination (R1 contained the cells of interest and contained not fewer than 7000 events). Median fluorescence values for SPE and cytokeratin FITC were measured. A further gate was set on the cytokeratin positive cell population (3% positive gate on tube 2 control) of cells (R2) and the median fluorescence value obtained for those cells in both gates (R1*R2). The R1*R2 gate excludes debris (including cytokeratin-positive debris) and those whole cells which were cytokeratin negative. This gate contained on average 5000 events. No sample was considered if fewer than 1000 events were present. Median fluorescence values were converted into binding capacities using Quickcal, QSC calibration software with correction for non-specific binding.

### Results

Fifty-three confirmed solid breast cancers and 33 FNA samples were collected from 66 patients. Of these, 20 paired samples were suitable for study. Reduction in the numbers of available samples was due to failure to sample during surgery or lack of tissue/cells during pathological examination.

*Cytokeratin staining*

All samples were assessed for cytokeratin staining. The data were examined as a percentage of positive cells and as number of cytokeratin molecules per cell. For the 53 solid cancer specimens the mean percentage of cytokeratin-positive cells was 64% (s.e.m. ± 3.49). Examination of the FNA group showed a mean value of 43% (s.e.m. ± 6.03). Statistical analysis by Student's *t*-test showed these to be significantly different ($P = 0.004$) with higher proportions of non-epithelial cells in the FNAs. Examination of the same data in terms of the number of molecules of cytokeratin per cell showed no significant difference ($P = 0.88$). FNA samples showed a range of between $5 \times 10^2$ and $3 \times 10^8$ molecules per cell (mean = $1 \times 10^7$, s.e.m. $\pm 9 \times 10^6$) while solid tumours showed between $4 \times 10^3$ and $2 \times 10^8$ molecules per cell (mean = $1 \times 10^7$, s.e.m. $\pm 4 \times 10^6$).

The cytokeratin data on paired samples only were then examined. Using Student's paired t-test cytokeratin content was observed for each of the paired samples. FNAs showed a mean value of 55.12% (s.e.m. ± 7.42) and the solid tumours 66.53% (s.e.m. ± 5.37); the result was not significantly different ($P = 0.51$). Examination of the number of molecules of cytokeratin on paired data again was not significantly different ($P = 0.2$) with FNAs showing a mean value of $2 \times 10^7$ (s.e.m. $\pm 1.6 \times 10^7$) molecules and the solid tumours $3 \times 10^7$ (s.e.m. $\pm 9 \times 10^5$).

### *Oestrogen receptor staining*

ER was also measured on all solid and FNA samples. ER levels were calculated in terms of number of molecules. Examination of the tumour alone, without cytokeratin gating, showed a mean value of 3815 (s.e.m. ± 715) molecules of ER per cell compared with 5746 (s.e.m. ± 932) when cytokeratin gating was performed (Figure 1a). A strong correlation was observed between these data ($P < 0.00001$, $r^2 = 0.71$). Figure 1b illustrates the relationship between ER levels on FNA samples with a mean value of 96 222 (s.e.m. ± 90 747) molecules with and 2360 (s.e.m. ± 1102) molecules without application of a cytokeratin gate ($P = 0.0002$, $r^2 = 0.40$). It was noted that the FNA expressing the highest level of ER in the cytokeratin-gated population significantly increased the mean value, which is somewhat misleading. The median value for this population was 4804 molecules per cell.

Twenty paired samples of FNA and tumour were examined for ER expression. In the absence of a cytokeratin gate a slight correlation was observed ($P = 0.06$, $r^2 = 0.18$, Figure 1c). ER expression levels varied between 1 and 16 315 (mean 2741, s.e.m. ± 995) molecules for the tumours and between 1 and 9203 (mean 1854, s.e.m. ± 530) molecules for the FNAs. FNAs showed lower levels of ER expression than the tumours. Cytokeratin gating these same samples showed ER expression levels varying between 181 and 27 444 (mean 5726, s.e.m. ± 1321) molecules for the tumours and between 1 and 27 047 (mean 4252, s.e.m. ± 1412) molecules for the FNAs. The cytokeratin gating has brought the mean ER expression for both FNA and solid tumour closer together. The gate clearly affects the distribution pattern of ER expression and a correlation ($P = 0.005$, $r^2 = 0.40$) was observed (Figure 1d).

### *c-erbB-2 staining*

Thirteen paired samples of FNA and tumour were examined for c-erbB-2 expression. In the absence of a cytokeratin gate no correlation was observed ($P = 0.2$, $r^2 = 0.10$). Expression of c-*erb*B-2 levels varied between 186 and 22 115 (mean 4726, s.e.m. ± 1693) molecules for the tumour and between 305 and 12 021 (mean 3632, s.e.m. ± 909 molecules) for the FNA. The results are summarised in Figure 2a and show no clear distribution pattern. Indeed, the regression line is so bad as to be meaningless; it has been left in merely to illustrate this point. However, in Figure 2b we show that employment of a cytokeratin gate clearly affects the distribution pattern of c-erbB-2 expression and a slight correlation can be observed ($P = 0.05$, $r^2 = 0.30$). Expression of c-erbB-2 levels varied between 252 and 32 037 (mean 5505, s.e.m. ± 2756) molecules for the tumour and between 865 and 9084 (mean 4051, s.e.m. ± 785) molecules for the FNA.

### *EGFR staining*

Thirteen paired samples of FNA and tumour were examined for EGFR expression. In the absence of a cytokeratin gate no correlation was observed ($P = 0.21$, $r^2 = 0.14$). EGFR expression levels varied between 209 and 15 428 (mean 2450, s.e.m. ± 1126) molecules for the tumours and between 1 and 9956 (mean 1626, s.e.m. ± 757) molecules for the FNAs. The results are summarised in Figure 3a. However, employment of a cytokeratin gate, like that for c-erbB-2, clearly affects the distribution pattern for EGFR expression and a correlation

**Figure 1** (**a**) The linear regression correlation for ER expression on 53 solid tumour specimens with and without the employment of a cytokeratin gate. (**b**) The correlation for ER expression on 33 FNA specimens with and without the employment of a cytokeratin gate. (**c**) The lack of correlation between FNA and solid tumour expression of ER without the use of a cytokeratin gate. (**d**) The effect of cytokeratin gating on these samples.

**Figure 2** (**a**) The lack of correlation between FNA and solid tumour expression of c-*erb*B-2 without the use of a cytokeratin gate. (**b**) The effect of cytokeratin gating on these samples.

**Figure 3** (**a**) The lack of correlation between FNA and solid tumour expression of EGFR without the use of a cytokeratin gate. (**b**) The effect of cytokeratin gating on these samples.

was observed ($P = 0.05$, $r^2 = 0.3$, Figure 3b). EGFR expression levels varied between 186 and 22 115 (mean 4726, s.e.m. = 1693) molecules for the tumours and between 305 and 12 021 (mean 3632, s.e.m. ± 909) molecules for the FNAs.

### *Examination of ploidy*

Examination of ploidy was carried out on all 20 paired tumour/FNA samples. In all cases the ploidy of FNAs was the same regardless of cytokeratin gating. Ploidy of the solid tumour was constant except for one case in which cytokeratin gating revealed an aneuploid phenotype (near diploid) which was classed as diploid previous to gating. However, comparison of FNAs and solid tumours showed some marked differences (denoted by an asterisk in Table I). Two cases of missed aneuploids in the FNA group were clearly revealed; on both occasions the tumour sample was aneuploid when the FNA was diploid.

### Discussion

This paper set out to ask the question 'Are fine-needle aspirates representative of the tumour from which they were taken?' Examination of FNA samples by flow cytometry is influenced by several factors and several potential problems occur.

The most immediate problem identified has been obtaining enough cells to perform an adequate flow cytometric analysis. This is reflected in the reduced numbers of paired data compared with individual solid tumour numbers. The same problem has been identified during cytological diagnosis (Brown *et al.*, 1991) with a small proportion of samples containing no epithelial cells. The use of multiple aspirates has alleviated this problem to some extent but leads to two

**Table I** Effect of cytokeratin gating on ploidy assessment

| | *FNA* | | *Solid tumour* | |
|---|---|---|---|---|
| *Patient No.* | *All cells* | *$CK^+$ cells* | *All cells* | *$CK^+$ cells* |
| 1 | Tetraploid | Tetraploid | Tetraploid | Tetraploid |
| 2 | Diploid | Diploid | Diploid | Diploid |
| 3 | Aneuploid | Aneuploid | Aneuploid | Aneuploid |
| 4 | Aneuploid | Aneuploid | Aneuploid | Aneuploid |
| 5 | Diploid | Diploid | Diploid | Aneuploid[a] |
| 6 | Diploid | Diploid | Diploid | Diploid |
| 7 | Tetraploid | Tetraploid | Tetraploid | Tetraploid |
| 8 | Diploid | Diploid | Aneuploid[a] | Aneuploid[a] |
| 9 | Diploid | Diploid | Diploid | Diploid |
| 10 | Diploid | Diploid | Aneuploid[a] | Aneuploid[a] |
| 11 | Tetraploid | Tetraploid | Tetraploid | Tetraploid |
| 12 | Diploid | Diploid | Diploid | Diploid |
| 13 | Diploid | Diploid | Diploid | Diploid |
| 14 | Diploid | Diploid | Diploid | Diploid |
| 15 | Diploid | Diploid | Diploid | Diploid |
| 16 | Diploid | Diploid | Diploid | Diploid |
| 17 | Diploid | Diploid | Diploid | Diploid |
| 18 | Diploid | Diploid | Diploid | Diploid |
| 19 | Aneuploid | Aneuploid | Aneuploid | Aneuploid |
| 20 | Aneuploid | Aneuploid | Aneuploid | Aneuploid |

[a]Denotes the presence of missed aneuploid peaks.

further problems. Increased numbers of cells do not necessarily mean that tumour cells are present. Cytological observation is still recommended at this stage to determine if all epithelial cells are normal.

Furthermore, increased sampling leads to increased levels of contaminating cells. Red cell contamination is combated by the use of saponin, which causes leakage of haemoglobin and loss of red cell side-scatter signal. The remaining cells can be further 'purified' by use of cytokeratin gating to select all epithelial cells.

Cytokeratin levels have been measured on many tumours (Angus et al., 1987; Ferrero et al., 1990; Brotherick et al., 1994b) and have been shown to be of importance in gating out debris and contaminating stromal and lymphoid cells (Ferrero et al., 1990; Brotherick et al., 1995). Such studies have relied on high proportions of tumour cells being present. However, cytokeratin also labels benign epithelial cells. In this study we have shown cytokeratin levels for FNAs and solid tumour samples are correlated when the number of molecules are compared. Interestingly, however, analysis of the percentage of positive cells showed a significant difference between FNAs and solid samples. Whether this is a good reason for abandoning the percentage of positive in favour of number of molecules is not clear. Observations of true *paired* data showed no difference between aspirate and tumour sample for percentage of positive or number of molecules of cytokeratin.

Cytokeratin levels are usually high in solid samples of breast tumours. Such a well-expressed marker would be expected to occur at a detectable level in FNA samples, resulting in a correlation between FNAs and solid tumours. However, in the cases of ER and c-*erb*B-2, where methodological constraints can result in poor marker detection, contaminating signals are likely to cause spurious results. We have shown this to be true in the case of ER expression (Brotherick et al., 1995), in that non-epithelial cells which are oestrogen receptor negative cause a reduction in overall fluorescence with subsequent difficulty in determining the two populations from a fluorescence histogram. Low cell numbers in FNA samples cause little correlation between FNA and tumour (Figures 1c, 2b and 3b), especially when no cytokeratin gate is present. Cytokeratin gating may reduce the number of cells measured but enhances the correlation between tumour and FNA sample. The problem may therefore be due not to low cell numbers but rather to the effect that non-epithelial cells have on the fluorescence signal.

For this reason ER, EGFR and c-erbB-2 were examined with and without cytokeratin gating. ER has already been reported to be poorly expressed in breast tumours and cytokeratin gating has been shown to be of importance in measuring ER expression in solid tumours (Brotherick et al., 1995). The level of EGFR on both breast and bladder cells is usually of the order of $1 \times 10^4$ molecules per cell (Brockhof et al., 1994; Brotherick et al., 1994a). Expression of c-*erb*B-2 has been measured by flow cytometry (Stål et al., 1994). However c-erbB-2 expression, in terms of number of molecules, has not been reported, although from our work we have shown that around 3200 molecules per cell appears to be the threshold above which positive cancers are determined (Brotherick et al., 1994b).

On examining the total tumour population alone, with and without cytokeratin gating, a good correlation for ER expression was found. Similarly, on the same aspirate a close correlation for ER expression with or without cytokeratin gating was seen. This reflects the reproducibility of the analysis performed. However, when paired samples were analysed we found that cytokeratin gating was important for the FNA to be representative of the solid tumour. This finding is reflected in Figures 1c and d. EGFR and c-erbB-2 expression both followed this pattern (Figures 2 and 3), with no observable correlation for either, unless a cytokeratin gate was used. These findings indicate that FNA sampling is only representative of the solid tumour if a cytokeratin gate is used.

Examination of ploidy has shown that aspirate and solid tumour findings generally agree. In most cases abnormal DNA histograms were clearly identifiable and cytokeratin gating merely clarifies such peaks and also aids in the measurement of cell cycle phases. However, two FNA samples failed to show the presence of an aneuploid population of cells which was picked up during examination of the solid tumour even without the use of a cytokeratin gate. This finding reflects the limitation in cell numbers to give a reasonable DNA profile which can be easily analysed. The aneuploid populations were clearly visualised with the aid of cytokeratin gating. Alteration in ploidy as a result of regional intratumoral variation was not considered to be a problem because of the rigorous protocol for aspiration. It is reasonable to conclude that DNA profiles need more cells to allow analysis of small aneuploid populations. Solid tumour samples have ample aneuploid cells within the total cell population and small aneuploid populations are relatively easy to identify. The presence of cytokeratin-positive normal epithelial cells can further hinder aneuploid detection. High levels of cytokeratin expression in FNA can mask the presence of aneuploid populations. After gating one tumour (which has been identified as diploid without cytokeratin gating) it was correctly identified as aneuploid. In this case cytokeratin gating was useful in identifying the abnormal cells. These findings fit in with those of Ferrero et al. (1990), who have assessed the use of cytokeratin staining in the assessment of DNA content.

Use of FNA samples of breast cancers, while presenting some problems, does not appear to be representative of the underlying solid tumour. Despite the lower levels of detection of ER, EGFR and c-erbB-2, these problems are surmountable by the use of regulated cut-off points above which positivity of a tumour is determined. Such levels would need to be assessed in a large prospective study before they could be applied to clinical samples. The methodology would allow rapid detection of markers on breast tumours as near to the time of clinical presentation and cytological diagnosis as possible.

**Acknowledgements**
The authors would like to acknowledge Professor CHW Horne, Dr B Angus and Mr R Johnstone (Novacastra Laboratories) for 5D3, EGFR and c-erbB-2 antibodies and M Broe (Dako) for ER antibody. We also wish to thank the North of England Cancer Research Campaign for financial support.

**References**

ANGUS B, PURVIS J, STOCK D, WESTLEY BR, SAMSON ACR, ROUTLEDGE EG, CARPENTER FH AND HORNE CHW. (1987). A new monoclonal antibody recognising low molecular weight cytokeratins effective for immunohistochemistry using formalin-fixed paraffin embedded tissue. *J. Pathol.*, **153**, 377–384.

BERGER MS, LOCHNER GW, SAURER S, GULLICK WJ, WATERFIELD MD, GRONER, B AND HYNES NE. (1988). Correlation of the c-erbB-2 gene amplifications and protein expression in human breast carcinoma with nodal status and nuclear grading. *Cancer Res.*, **48**, 1238–1243.

BROCKHOFF G, HOFSTAEDER F AND KNUECHEL R. (1994). Flow cytometric detection and quantitation of the epidermal growth factor receptor in comparison to Scatchard analysis in human bladder carcinoma cell lines. *Cytometry*, **17**, 75–83.

BROWN LA, COGHILL SB AND POWIS AJ. (1991). Audit of diagnostic accuracy of FNA cytology specimens taken by the histopathologist in a symptomatic breast clinic. *Cytopathology*, **2**, 1–6.

BROTHERICK I, LENNARD TWJ, WILKINSON SE, COOK S, ANGUS B AND SHENTON BK. (1994*a*). Flow cytometric method for the measurement of epidermal growth factor receptor and comparison with the radio-ligand binding assay. *Cytometry*, **16**, 262–269.

BROTHERICK I, COWAN WK, HIGGS M, ANGUS B, HORNE CHW, LENNARD TWJ AND SHENTON BK. (1994*b*). Association between c-erbB-2 proto-oncogene expression, grade and ploidy in breast tumours (abstract). *Cytometry*, **7** (Suppl.), 78.

BROTHERICK I, LENNARD TWJ, WILKINSON SE, COOK S, ANGUS B, WINTHEREIK MP AND SHENTON BK. (1995). Use of the biotinylated antibody Dako-ER 1D5 to measure oestrogen receptor on cytokeratin positive cells obtained from primary breast cancer cells. *Cytometry*, **20**, 74–80.

FERRERO M. SPYRATOS F. LE DOUSSAL V. DESPLACES A AND ROUËSSÉ J. (1990). Flow cytometric analysis of DNA content and keratins by using CK7, CK8, CK18, CK19, and KL1 monoclonal antibodies in benign and malignant human breast tumors. *Cytometry*, **11**, 716–724.

GULLICK WJ. LOVE SB. WRIGHT C. BARNES DM. GUSTERSON B. HARRIS AL AND ALTMAN DG. (1991). c-erbB-2 protein overexpression in breast cancer is a risk factor in patients with involved and uninvolved lymph nodes. *Br. J. Cancer*, **63**, 434–438.

HOWELL A. BARNES DM. HARLAND RN. REDFORD J. BRAMWELL VH AND WILKINSON MJ. (1984). Steroid-hormone receptors and survival after first relapse in breast cancer. *Lancet*, **1**, 588–591.

NICHOLSON S. SAINSBURY JRC. NEEDHAM GK. CHAMBERS P. FARNDON JR AND HARRIS AL. (1988). Quantitative assays of epidermal growth factor receptor in human breast cancer: cutoff points of clinical relevance. *Int. J. Cancer*, **42**, 36–41.

PAIK S. HAZAN R. FISHER ER. SASS RE. FISHER B. REDMOND C. SCHLESSINGER J. LIPPMANN ME AND KING CR. (1990). Pathological findings from the national surgical adjuvant breast and bowel project: prognostic significance of erbB-2 protein overexpression in primary breast cancer. *J. Clin. Oncol.*, **8**, 103–112.

PERREN TJ. (1991). c-erbB-2 oncogene as a prognostic marker in breast cancer. *Br. J. Cancer*, **63**, 328–332.

POWLES TJ. TROTT PA. CHERRYMAN G. CLARKE S. ASHLEY S. COOMBES RC. JONES AL. SINNETT HD AND NASH AG. (1991). Fine needle aspiration cytodiagnosis as a pre-requisite for primary medical treatment of breast cancer. *Cytopathology*, **2**, 7–12.

SAINSBURY JRC. MALCOLM AJ. APPLETON DR. FARNDON JR AND HARRIS AL (1985). Presence of epidermal growth factor receptor as an indicator of poor prognosis in patients with breast cancer. *J. Clin. Pathol.*, **38**, 1225–1228.

SLAMON DJ. CLARK GM. WONG SG. LEVIN WJ. ULLRICH A AND McGUIRE WL. (1987). Human breast cancer: correlation of relapse and survival with amplification of HER 2neu oncogene. *Science*, **235**, 177–182.

STÅL O. SULLIVAN S. SUN X-F. WINGREN S AND NORDENSKJÖLD B. (1994). Simultaneous analysis of c-erbB-2 expression and DNA content in breast cancer using flow cytometry. *Cytometry*, **16**, 160–168.

WHITESIDE TL. MIESCHER S. HURLIMANN J. MORETTA L AND VON FLIEDNER V. (1986). Clonal analysis and in situ characterisation of lymphocytes infiltrating human breast carcinomas. *Cancer Immunol. Immunother.*, **23**, 169–178.

WRIGHT C. ANGUS B. NICHOLSON S. SAINSBURY JRC. CAIRNS J. GULLICK WJ. KELLY P. HARRIS AC AND HORNE CHW. (1989). Expression of c-erbB-2 oncoprotein: a prognostic indicator in human breast cancer. *Cancer Res.*, **49**, 2087–2090.

YUAN J. HENNESSEY C. CORBETT IP. DYKIN R. GIVAN AL. SHENTON BK. HENRY JA. WRIGHT C AND LENNARD TWJ. (1991). Node negative breast cancer: the prognostic value of DNA ploidy for long-term survival. *Br. J. Surg.*, **78**, 844–848.